# Reply to ‘Beyond socioeconomic status: structural determinants and strategic approaches to regional variation in left ventricular assist device utilization’

**DOI:** 10.1007/s12471-026-02046-6

**Published:** 2026-05-08

**Authors:** Valérie C. E. Drost, Maaike Wösten, Luuk C. Otterspoor, Kadir Caliskan, Linda W. Van Laake

**Affiliations:** 1 Thoraxcenter, Department of Cardiology, Cardiovascular Institute, Erasmus MC University Medical Center, Rotterdam, The Netherlands; 2 Department of Cardiology, Catharina Hospital, Eindhoven, The Netherlands; 3 Department of Biomedical Engineering, Eindhoven University of Technology, Eindhoven, The Netherlands; 4 Department of Cardiology and Transplantation Center Utrecht, University Medical Center Utrecht, Utrecht, The Netherlands; 5 Computational Imaging Group for MRI Therapy & Diagnostics, University Medical Center Utrecht, Utrecht, The Netherlands; 6 Utrecht University, Utrecht, The Netherlands

Dear Editor,

We thank the authors for their interest in our manuscript and for sharing their experiences from Japan. Beyond our recent study findings, LVAD utilization—as well as timely referral and sustained patient management—may indeed also be influenced by regional variations in organization and coordination in the Dutch context. On the other hand, institutional capacity has not been a limiting factor thus far [1].

National education programs for cardiologists (e.g. CVOI—‘Masterclass Heart Failure’) and regional outreach programs for referring hospitals are organized. In the absence of a uniform model with formalized referral pathways, informal referral structures are predominantly used in the Netherlands. In 2022, the first formal LVAD shared-care program was established in the south, by Erasmus University Medical Center as the implanting center and Catharina Hospital as the follow-up center [2].

To conclude, our recent publication points to the need for improvement in delivering LVAD therapy to eligible patients. Alongside developing a national model, further gains may be achieved by strengthening recognition of advanced heart failure and optimizing referral pathways [3].

## Data Availability

Data sharing is not applicable to this article as no datasets were generated or analysed.
